# Defining a role for non-satellite stem cells in the regulation of muscle repair following exercise

**DOI:** 10.3389/fphys.2013.00310

**Published:** 2013-11-05

**Authors:** Marni D. Boppart, Michael De Lisio, Kai Zou, Heather D. Huntsman

**Affiliations:** Department of Kinesiology and Community Health, Beckman Institute for Advanced Science and Technology, University of IllinoisUrbana, IL, USA

**Keywords:** satellite cells, pericytes, SP cells, fibro/adipogenic progenitors, mesenchymal stem cells, eccentric exercise

## Abstract

Skeletal muscle repair is essential for effective remodeling, tissue maintenance, and initiation of beneficial adaptations post-eccentric exercise. A series of well characterized events, such as recruitment of immune cells and activation of satellite cells, constitute the basis for muscle regeneration. However, details regarding the fine-tuned regulation of this process in response to different types of injury are open for investigation. Muscle-resident non-myogenic, non-satellite stem cells expressing conventional mesenchymal stem cell (MSC) markers, have the potential to significantly contribute to regeneration given the role for bone marrow-derived MSCs in whole body tissue repair in response to injury and disease. The purpose of this mini-review is to highlight a regulatory role for Pnon-satellite stem cells in the process of skeletal muscle healing post-eccentric exercise. The non-myogenic, non-satellite stem cell fraction will be defined, its role in tissue repair will be briefly reviewed, and recent studies demonstrating a contribution to eccentric exercise-induced regeneration will be presented.

## Muscle injury and repair post-exercise

Human movement is largely voluntary, requiring the conscious activation of the appropriate number of motor neurons necessary for muscle contraction. Shortening of the individual sarcomeres within skeletal muscle, also known as concentric contractions, are necessary for the transfer of force from muscle to the body's lever system. When the load placed on muscle is greater than the tension that can be created within the sarcomere, the muscle will initially resist lengthening by increasing the bond strength within the actin-myosin cross-bridge, but eventually surrender to movement, resulting in an eccentric contraction. Eccentric contractions are present predominantly during participation in planned resistance exercise; however, they also commonly occur during activities of daily living such as lifting and lowering heavy items or walking downstairs. While repeated shortening of muscle can induce long-term adaptations that allow for increased endurance and fatigue resistance, exercise that continually engages the muscle in an eccentric manner can increase sarcomere myofibrillar content, sarcomere number, and ultimately increase the muscle's ability to generate force. Thus, delineating the early events that occur with an acute bout of exercise that contribute to such beneficial adaptations can be informative in designing therapeutic interventions to improve the rate and efficiency of skeletal muscle healing following injury.

Eccentric contractions result in ultrastructural damage in both animal models and humans, with the degree of damage (mild, moderate, severe) dependent on several factors, including the method of stimulation (downhill running vs. electrical stimulation), the specific muscle used for evaluation, the indice of damage (direct vs. indirect markers), and the time course for observation (Friden et al., 1983; Newham et al., 1983; McCully and Faulkner, 1985; Smith et al., 1997; Lovering and Brooks, 2013). Muscle injury can initiate a repair process that is necessary for maintenance of tissue structure and preservation of function (Sambasivan et al., 2011). Information regarding the mechanistic basis for muscle repair following injury is predominantly obtained from studies which utilize barium chloride (BaCl_2_), cardiotoxin (CTX), notexin, bupivacaine and cryolesion as means of ablating muscle tissue in rodents. Whether the events that ensue following this extreme approach to studying muscle damage reflect the precise adaptations that occur post-exercise has not been established. Despite this limitation, the accepted model for muscle regeneration in response to injury includes an extended inflammatory response, including activation of resident macrophages, immediate recruitment of neutrophils (1–2 h), macrophage infiltration (12–24 h), M1 (proinflammatory, phagocytic) to M2 (anti-inflammatory) macrophage polarization (24–48 h), and proliferation and activation of the primary progenitor cell in muscle, the satellite cell (1–8 days). For a more extensive review regarding a role for the immune system in repair and commentary regarding a role for inflammation in the regenerative response to exercise, refer to Saclier et al. (2013). In addition, Murphy et al. (2011) provides an interesting perspective regarding a role for fibroblasts in the regulation of satellite cell proliferation and differentiation.

Satellite cells, Pax7^+^ progenitor cells located in the niche between the sarcolemma and the basal lamina, become activated (expressing myogenic regulatory factors *Myf5* and *MyoD*), transiently proliferate and upregulate genes necessary for terminal differentiation (*myogenin* and *MRF4*) in response to injury (Charge and Rudnicki, 2004), including exercise-induced injury (Armand et al., 2003; Kadi et al., 2005; Cermak et al., 2013). The developing myoblast can fuse with a fiber, allowing for routine maintenance of myonuclei, or replace myonuclei lost due to sporadic injury or immobilization (Charge and Rudnicki, 2004). The recent development of sophisticated genetic tools to temporally eliminate the Pax7^+^ cell population in adult muscle corroborate previous evidence that satellite cells are essential for muscle regeneration following both chemical and exercise/load-induced muscle injury (McCarthy et al., 2011; Murphy et al., 2011; Sambasivan et al., 2011; Relaix and Zammit, 2012). While diphtheria toxin A–mediated cell elimination models do have limitations, these studies provide evidence that *direct* contribution of other cells residing in the interstitium or cells recruited from a distal source such as bone marrow is minimal. Thus, current studies focus on understanding the cues within the microenvironment that influence satellite cell renewal and differentiation, including the composition and milieu secreted by both inflammatory and non-inflammatory cells.

## Mesenchymal stem cells contribute to the repair of multiple tissues

Mesenchymal stem/stromal cells (MSCs), first discovered in 1968 by Friedenstein et al. (1968), are a multipotent cell population located in a number of tissues throughout the body. Their identity as a stem cell, determined by their self-renewal capacity, is still controversial; therefore, the term mesenchymal stem cell is reserved for those cells that have demonstrated this capacity while all other MSC populations are termed mesenchymal stromal cells (Keating, 2006). MSCs are generally defined functionally as no single cellular marker is available for isolation of a pure MSC population. According to the International Society for Cellular Therapy, human MSCs are spindle-shaped, plastic adherent, positive for the cell surface markers CD105, CD73, CD90, negative for the cell surface markers CD45, CD34, CD14, or CD11b, CD79 or CD19, and HLA-DR, and are multipotent in that they can be induced to differentiate along the osteogenic, adipogenic and chondrogenic lineages (Dominici et al., 2006). Their multipotency initially led to great excitement for the use of MSC in cell therapy. This excitement was burgeoned when it was discovered that MSC are immunoprivileged and do not elicit an immune response in their new host suggesting that cell therapy using MSC could be safe and viable (Keating, 2006). Indeed, using pre-clinical models, MSC therapy has been demonstrated to enhance wound healing (Dantzer et al., 2003), accelerate regeneration following spinal cord injury (Mansilla et al., 2005), and increase heart repair following myocardial infarction (Ranganath et al., 2012), among others.

Although it was initially hypothesized that enhanced regeneration provided by MSC therapy was due to replacement of lost or damaged cells by MSC differentiation, current studies highlight MSC stromal support as a primary mechanism for regeneration (Ranganath et al., 2012). Indeed MSCs have been demonstrated to synthesize and release factors including, but not limited to, vascular endothelial growth factor (VEGF), fibroblast growth factor-2 (FGF-2), hepatocyte growth factor (HGF) and insulin-like growth factor-1 (IGF-1) (Gnecchi et al., 2006). The prevailing hypothesis is that MSCs release these paracrine factors locally, or systemically, in response to unidentified factors released by the injured microenvironment and that these events are necessary to efficiently promote tissue regeneration.

## Non-satellite stem cells express MSC markers and enhance skeletal muscle repair

Side population (SP) cells, mesenchymal progenitors, pericytes, muscle-derived stem cells, fibro/adipogenic progenitors (FAPs), and interstitial stem cells (PW1^+^) provide the nomenclature for Pax7 negative, multipotent mononuclear cells residing in muscle (Qu-Petersen et al., 2002; Motohashi et al., 2008; Joe et al., 2010; Mitchell et al., 2010; Uezumi et al., 2010; Doyle et al., 2011; Valero et al., 2012; Wosczyna et al., 2012). Despite the fact that the majority of these cell types express MSC markers and exhibit multi-lineage potential, investigators are hesitant to designate them as MSCs. This is partly due to the strict guidelines necessary to confirm MSC status and lack of consensus regarding the precise markers used for identification. Whether the non-satellite stem cell fraction in muscle represent slightly modified versions of a primary mesodermal stem cell or whether these cells are unique descendants with distinct phenotypes is not known. Regardless, one trait that underlies all non-satellite stem cells in muscle is their ability to expand within the interstitium in response to muscle fiber injury. Here we evaluate the contribution of non-satellite stem cells to repair post-injury, presenting only studies that have established a role for non-myogenic, non-satellite stem cells in repair following chemical injury.

### Side population (sp) cells

SP cells, first identified in the bone marrow based on Hoechst 33342 dye exclusion, were reported to be present in muscle and contribute to both muscle and vascular regeneration following injury (Gussoni et al., 1999; Asakura et al., 2002; Majka et al., 2003). While the majority of muscle SP cells were identified as CD31^+^ endothelial cells, a fraction of muscle-derived SP cells negative for CD31 (CD31^−^CD45^−^) were found to proliferate and contribute to new fiber formation in response to CTX injection (Uezumi et al., 2006). CD31^−^CD45^−^ SP cells extracted from regenerating muscle not only expressed several mesodermal-mesenchymal genes post-injury, such as platelet-derived growth factor receptor α (PDGFRα), but also demonstrated the unique capacity to spontaneously differentiate into adipocytes or form osteogenic cells in the presence of osteogenic media *in vitro*. In addition, CD31^−^CD45^−^ SP cells retrieved from injured muscle expressed angiogenic factors [e.g., angiopoietin-1 (ang-1) and VEGF] and tumor growth factor beta (TGF-β) antagonists (e.g., follistatin). Thus, the importance of this study was the identification of a sub-fraction of SP cells that could act as tissue-resident MSCs and directly and indirectly contribute to muscle repair post-injury.

Despite the suggestion that CD31^−^CD45^−^ SP cells could directly contribute to new fiber synthesis post-injury, such potential was subsequently found to be limited. Motohashi and colleagues determined that CD31^−^CD45^−^ SP cells do not readily become muscle, but rather enhance transplantation and proliferation of exogenously injected myoblasts and increase growth of myoblast-engrafted fibers following CTX injection (Motohashi et al., 2008). Further gene expression profiling suggested that SP cells synthesize a wide variety of paracrine factors, including numerous factors known to promote muscle repair. Specifically, metalloproteinase-2 (MMP-2) was highlighted as one factor that could be released and promote myoblast migration following injury.

Doyle and colleagues recently evaluated SP cell fate using an inducible reporter for abcg2 (Abcg2^*CreERT2*^ × Rosa26-LacZ mice) (Doyle et al., 2011). LacZ^+^ cells accumulated in the interstitium of muscle, minimally fused with pre-existing fibers, and gave rise to a variety of cell types, including cells expressing stem cell antigen-1 (Sca-1) and the pericyte marker, PDGFRβ. Mice deficient in the expression of abcg2, thus lacking SP cells, displayed impaired regeneration following CTX injection.

Altogether, these studies suggest that SP cells, predominantly those that express PDGFRα and β and are negative for CD31 or CD45, are mesenchymal-like stem cells and/or pericytes which indirectly contribute to repair post-injury.

### PDGFRα^+^ progenitors (mesenchymal progenitors, FAPs, PICs)

Muscle-derived CD31^−^CD45^−^ non-satellite stem cells strongly express PDGFRα and vimentin, markers associated with undifferentiated MSCs (Uezumi et al., 2010). In a follow up study to their 2006 publication, Uezumi and colleagues isolated CD31^−^CD45^−^cells positive for PDGFRα^+^ from muscle and evaluated their capacity to differentiate into muscle *in vitro* and *in vivo*. CD31^−^CD45^−^PDGFRα^+^ cells did not demonstrate the capacity to become myogenic, but rather the majority (over 90%) acquired an adipogenic fate in culture. CD31^−^CD45^−^PDGFRα^+^ cells derived from GFP transgenic mice were traced and similarly became adipogenic following injection into muscles of WT mice exposed to glycerol; however, the same response did not occur following CTX injection. While CD31^−^CD45^−^PDGFRα^+^ cells rapidly expanded and did not differentiate into adipocytes following CTX injection, the role for these cells in muscle repair was not examined in this study.

The identification of a lin^−^α7 integrin^−^Sca-1^+^PDGFRα^+^ stem cell in muscle with adipogenic and fibrogenic potential in culture was similarly described and denoted fibro/adipogenic progenitors, or FAPs (Joe et al., 2010). Consistent with the Uezumi et al., 2010 study, FAPs significantly differentiated into adipocytes following glycerol injection, but this conversion did not occur in response to notexin. With notexin-mediated injury, FAPs did not undergo myogenesis or fuse with differentiating myogenic cells, yet were highly proliferative, localized to blood vessels and damaged myofibers, and secreted high levels of paracrine factor [IGF-1, interleukin-6 (IL-6), Wnt1, Wnt3A, Wnt5A]. FAP paracrine factor secretion may impact muscle repair, as FAPs markedly increased myoblast commitment to terminal differentiation as demonstrated in culture.

PW1^+^ interstitial cells (PICs) derived from muscle also express a broad range of genes common to MSCs and demonstrate multi-lineage potential (Pannerec et al., 2013). The extent to which PICs represent FAPs is not known, but substantial overlap in cell surface expression and function has been demonstrated in a subset of PICs expressing PDGFRα (Pannerec et al., 2013). The potential for PW1^+^PDGFRα^+^ cells to secrete pro-myogenic factors and repair tissue in response to unique stimuli has not been determined, but likely given the role for PW1 in the cellular response to stressors (Relaix et al., 1998).

Overall, the results from these studies suggest that muscle-resident PDGFRα^+^ mesenchymal progenitors may positively and indirectly contribute to regeneration, but such potential is regulated by both intrinsic and extrinsic factors. Determination of the predominant regulators of stem cell fate will be essential for capitalizing on FAP regenerative capacity, including the contribution to new fiber synthesis, the composition of the extracellular matrix and the immune system. One example is the demonstration that IL-4/IL-13 signaling can significantly inhibit FAP adipogenic conversion post-injury (Heredia et al., 2013).

### Pericytes

Pericytes are characterized by their distinct morphology, localization within the basement membrane of vessels, and expression of a unique panel of cell surface markers (NG2, CD146, PDGFRβ) (Crisan et al., 2008). Investigators have begun to delineate two fractions of NG2^+^ pericytes in muscle: type-1 characterized by negative expression for nestin and positive expression for PDGFRα (PDGFRβ ^+^CD146^+^Sca-1^+^CD34^+^Pax7^−^) and type-2 characterized by positive expression for nestin and negative expression for PDGFRα (PDGFRβ^+^CD146^+^Sca-1^−^CD34^−^Pax7^−^) (Birbrair et al., 2013). While both types are able to proliferate in response to glycerol or BaCl_2_-induced injury, type-1 pericytes give rise to adipogenic cells only in response to glycerol injection and type-2 become myogenic in response to both types of injury. The extent to which type-1 pericytes represent the mesenchymal progenitor or FAPs described, is not known (Joe et al., 2010; Uezumi et al., 2010). Regardless, both type-1 pericytes and FAPs have the potential to indirectly enhance myogenic progenitor differentiation (Joe et al., 2010; Birbrair et al., 2013).

It is now accepted that muscle-resident non-myogenic, non-satellite stem cells, potentially MSC descendants, accumulate in the interstitium following injury and their ability to fully contribute to repair is dependent on internal and external cues. From a muscle rehabilitation perspective, it would be interesting to determine whether these cells expand in muscle in response to exercise (resistance training, endurance exercise) or physical therapy and understand if the mechanisms that underlie their contribution to healing are the same as those that occur in response to chemical injury.

## Non-satellite stem cells contribute to regeneration post-exercise

The α7β1 integrin is a transmembrane heterodimeric protein that can link laminin in the extracellular matrix to the myoblast and myotube cytoskeleton for the purposes of cellular signaling, migration and adhesion (Crawley et al., 1997). We have previously demonstrated that a single 30 min bout of eccentric exercise can result in injury and upregulate transcription and protein expression of the α7 integrin subunit at 24 h post-exercise (Boppart et al., 2006, 2008). We subsequently determined that transgenic expression of the α7 integrin under a muscle-specific promoter (MCK:α7B integrin; α7Tg) can prevent eccentric exercise-associated damage and macrophage infiltration, while paradoxically stimulating a rapid increase in satellite cell number and new fiber synthesis (Lueders et al., 2011).

The identification of non-satellite stem cells in skeletal muscle with regenerative potential persuaded us to evaluate the presence of these cells in both wild type (WT) and transgenic mice post-eccentric exercise (Valero et al., 2012). Given the fact that Sca-1 is commonly expressed by mesenchymal progenitors, including those described above, positive selection for Sca-1 and negative selection for CD45 was chosen in an effort to maximally retrieve all potential non-myogenic, non-satellite stem cells following an acute bout of exercise. The percentage of Sca-1^+^CD45^−^ cells was increased 2-fold (4.3% at rest to 9.4% post-exercise) in WT muscle 24 h post-exercise and the total percentage was further enhanced with overexpression of the α7 integrin (8.7% at rest to 16.2%). The accumulation of Sca-1^+^CD45^−^ cells in muscle was dependent on the presence of the integrin or muscle integrity since this fraction was minimally present in α7 integrin null mice (Valero et al., 2012). Sca-1^+^CD45^−^ cells isolated from α7Tg muscle post-exercise were confirmed negative for Pax7 and were characterized by high level expression (>50%) for pericyte markers (NG2, CD146, PDGFRβ) and low expression (<1%) for endothelial markers (CD31, CD34). In addition, multi-lineage potential was established. Since these cells met the criteria for MSCs, including morphology, expression of MSC markers, and multi-lineage potential, they were designated muscle-derived MSCs, or mMSCs to distinguish them from MSCs derived from other tissue types, including bone marrow and adipose.

While mMSCs in our hands did not directly give rise to newly established fibers or vessels, they indirectly contributed to satellite cell expansion, new fiber synthesis, and vascular growth when transplanted into pre-exercised host limbs (Valero et al., 2012; Huntsman et al., 2013). We consistently find that mMSCs can only support regeneration when transplanted into muscle previously injured (1 h prior) by eccentric exercise. Thus, we speculate that mMSCs represent mesenchymal progenitors, FAPs, PW1^+^ and/or type-1 pericytes previously described (Joe et al., 2010; Birbrair et al., 2013) and provide a stromal role in tissue repair post-exercise (Valero et al., 2012).

We recently have established that mMSCs secrete a wide variety of beneficial growth factors and anti-inflammatory cytokines, including IGF-1, IL-6, VEGF, HGF, and epidermal growth factor (EGF) upon extraction from exercised muscle and the release of protein is additionally enhanced by application of mechanical strain in the absence of other cell types *in vitro* (Huntsman et al., 2013). These observations suggest that non-chemical cues, including strain and stiffness, dictate endogenous non-satellite stem cell fate and stromal release in response to exercise. The extent to which treatment of isolated non-myogenic, non-satellite stem cells with mechanical strain can alter the secretory milieu and improve engraftment upon injection into a fibrotic or less accommodating environment is a timely and worthwhile area of investigation.

We are only aware of one other study that has examined the non-satellite stem cell response to exercise. Hyldahl and colleagues reported the presence of mononuclear cells expressing pericyte markers [NG2 and alkaline phosphatase (AP)] in the interstitium of human skeletal muscle (Hyldahl et al., 2011) 3 h following a single bout of eccentric exercise. Although the number of cells remained unaltered at this early time point, NF-kB activity was significantly increased in NG2^+^ and ALP^+^ mononuclear cells. The significance of this event is not known, but likely reflects a role in differentiation or paracrine factor secretion.

**Figure 1:**
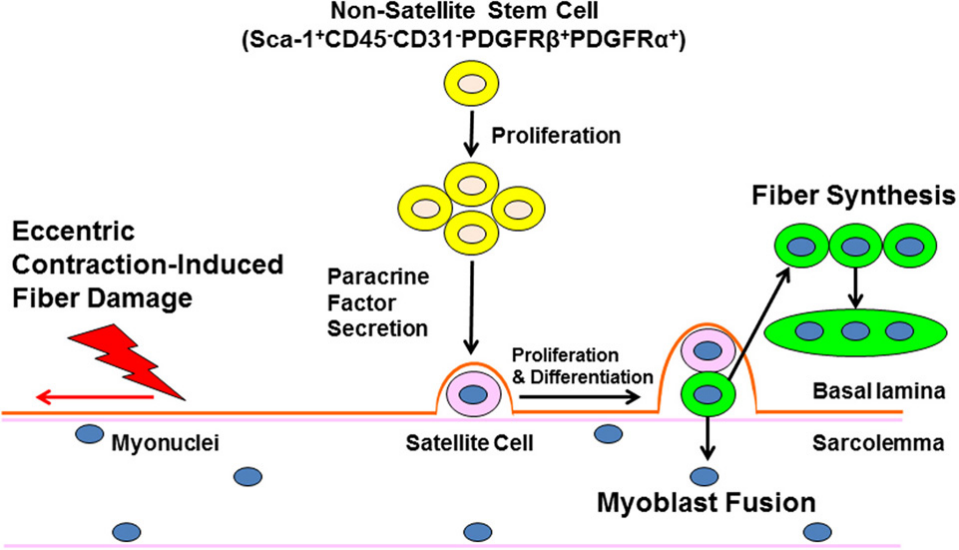
**Non-satellite stem cells regulate skeletal muscle repair following eccentric exercise.** Ultrastructural damage caused by eccentric exercise stimulates the expansion on non-satellite stem cells in skeletal muscle. Non-satellite stem cells synthesize and release a variety of growth factors and anti-inflammatory cytokines that positively regulate satellite cell proliferation and differentiation. Finally, developing myoblasts fuse with damaged fibers or fuse with other myoblasts to enhance new fiber synthesis. The regulation of non-satellite stem cell function by factors unique to different types of injury and conditions (age, disease) is a current area of investigation.

Information regarding the non-myogenic, non-satellite stem cell response to eccentric exercise is limited. The probability that that these cells are resistant to adipogenesis and/or fibrogenesis in a young, non-diseased individual post-exercise is high given the ability for eccentric exercise to elicit a rapid and effective repair process and mount an adaptive response which includes protection from further mechanical damage (repeat bout effect), maintenance of muscle mass, and enhanced force capacity (Figure 1). We predict that the unique mechanical and/or chemical cues provided by exercise alter the gene expression profile of the non-myogenic, non-satellite stem cell fraction in skeletal muscle such that the secretome can optimally support tissue function. Further studies are necessary to fully reveal the non-satellite stem cell response to exercise and this information will be important in the discovery of strategies to accelerate repair of damaged muscle and combat muscle loss with disease and age.

### Conflict of interest statement

The authors declare that the research was conducted in the absence of any commercial or financial relationships that could be construed as a potential conflict of interest.
